# Primary Large B-Cell Lymphoma of Immune-Privileged Sites of the Cerebellum: A Case Series and Review of the Literature

**DOI:** 10.3390/life13010201

**Published:** 2023-01-10

**Authors:** Saverio Pancetti, Daoud Rahal, Bethania Fernades, Carlo Galli, Silvia Uccella, Luigi Maria Terracciano, Federico Pessina, Lorenzo Bello, Arturo Bonometti

**Affiliations:** 1 Department of Biomedical Sciences, Humanitas University, Via Rita Levi Montalcini 4, Pieve Emanuele, 20090 Milan, Italy; 2 Department of Pathology Unit, Humanitas Clinical and Research Hospital, Via Manzoni 56, Rozzano, 20089 Milan, Italy; 3 Neurosurgery Department, IRCCS Humanitas Clinical and Research Hospital, Via Manzoni 56, Rozzano, 20089 Milan, Italy; 4 Neurosurgical Oncology Unit, Department of Oncology and Hemato-Oncology, IRCCS Galeazzi-Sant’Ambrogio, Università degli Studi di Milano, 20161 Milan, Italy

**Keywords:** diffuse large B-cell lymphoma, central nervous system, cerebellar, therapy, immune-privileged sites

## Abstract

Primary large B-cell lymphoma of immune-privileged sites (IP-LBCL) is a rare malignant hematological neoplasm. Involvement of the cerebellum is even rarer and its diagnosis is often difficult to make due to its non-specific clinical and radiological presentation. Methods: We reported 3 cases of cerebellar IP-LBCL followed at our hospital and reviewed the medical literature to unravel the peculiarities of this poorly studied entity. Outcomes: Analyzing our cases and reviewing the literature, we could collect and study 26 cases of cerebellar IP-LBCL. To the best of our knowledge, this is the largest cohort of such patients currently published. Conclusion: Cerebellar IP-LBCL presents more often in adult females with cerebellum-related focal neurological signs such as ataxia, headache, and nausea. Histological confirmation is mandatory for a correct diagnosis and treatment and all cases feature diffuse large B-cell lymphoma histopathology. Compared to other encephalic IP-LBCL, cerebellar cases seem to include a higher number of cases with germinal center B-cell phenotype and better survival. These differences may be related to a different immune microenvironment and especially immunoregulation that distinguishes the cerebellum from other areas of the CNS.

## 1. Introduction

According to the 5th edition of the WHO classification of lymphoid neoplasms, primary large B-cell lymphoma of immune privileged sites (IP-LBCL) is a rare B-cell lymphoma that arises in the central nervous system (CNS), vitreoretinal tissues, and testis of immunocompetent patients in their fifth to seventh decade. They roughly account for 3% of non-Hodgkin lymphoma (NHL) and 2–3% of all brain tumors [1,2,3]. From the category of IP-LBCL are excluded those lymphomas that arise in the same sites of immunodeficient patients or patients with immune dysregulation (including EBV, HCV, and HIV infection). The etiology of this lymphoma, as well as the possible cell of origin, is still completely unknown. IP-LBCL involving the encephalon (CNS) has a higher incidence among IP-LBCL. It more often presents with solitary lesions (65%) and involves the telencephalic hemispheres (38%) followed by the thalamus and basal ganglia (16%), corpus callosum (14%), periventricular region (12%) and posterior fossa/cerebellum (9%). Dural lymphomas are also excluded by definition from this category [4,5]. Clinical presentation of CNS IP-LBCL may vary, and often includes neurologic symptoms related to the mass-formin neoplasms and grossly specific to the involved region. Often before the diagnosis, the patients report a worsening of symptoms over weeks. Frequent symptoms include personality changes, changes in speech, focal neurological deficit, increased intracranial pressure, muscle weakness, seizures, ataxia, vomiting, confusion, and vision changes.

From a histopathological point of view, CNS IP-LBCL is diffuse large B-cell lymphomas (DLBCL) that display diffusely growing tumors with perivascular aggregates of medium-sized to large, atypical cells with nuclear pleomorphism and often prominent nucleoli and more or less extensive necrosis. The tumor proliferation elicits an intense astrocytic and microglial activation as well as a different degree of T-cell mediated response. The immunophenotype is that of a B-cell proliferation (PAX5+, CD20+, CD79a+) with immunoglobulin light chain restriction. Most cases (>90%) are CD10-negative and mostly display a non-germinal center B-cell phenotype (nGC) with positivity to Bcl6 and MUM1 in the vast majority of cases. Of interest, CD10 positivity should lead to suspect of a CNS spread of a systemic DLBCL. Mitotic figures are brisk and the proliferation index (Ki67/Mib1) is often as high as 80% [5]. From a molecular point of view, IP-LBCL carries rearranged and somatically hypermutated immunoglobulin genes. They show frequent rearrangements of the BCL6 gene, while translocations involving MYC or BLC2 genes are rare or absent. Half of the cases lose the expression of HLA class I and II proteins, explaining their ability to grow in immune privilege sites. Similarly, 50% of cases seem to harbor the MYD88 L265P mutation [5].

Another characteristic feature of IP-LBCL is their restricted homing to IP sites upon relapse. This may be also related to the lack of HLA proteins that protect neoplastic cells from immune activation against them. The prognosis is poor, and worse compared to other DLBCLs, with a median overall survival (OS) of 36 months. The treatment of choice in high-dose methotrexate-based polychemotherapy. Despite improving the outcome, the use of adjuvant whole-brain irradiation bears a high risk of neurotoxicity, especially in elderly patients. The rarity of IP-LBCL together with their non-specific even though potentially severe symptomatology may induce a delay in the final diagnosis [6].

Recently, regional heterogeneity in CNS immunity has been acknowledged. Specifically, the cerebellum seems to be different from other structures of the CNS in terms of blood–brain barrier permeability, cell trafficking, and immune responses [7]. We, therefore, wondered whether neoplastic proliferations of immune cells in the cerebellum—the most common being B-cell lymphomas—also have different features compared to those involving other CNS regions. In this article, we described three cases of cerebellar IP-LBCL followed in our center and gathered all described cases from the medical literature to compare their clinical, histopathological, and prognostic features. To the best of our knowledge, this is currently the most comprehensive work dealing with this subject.

## 2. Materials and Methods

### 2.1. Case 1

A 76-year-old Caucasian man presented with frontal headaches, dizziness, nausea, and vomiting. Magnetic resonance imaging (MRI) showed a mass in the left cerebellar hemisphere that while perfusion-weighted imaging underlined an elevated and abnormal blood flow in the cerebellar mass and a proton magnetic resonance spectroscopy (H-MRSI) confirmed a raise in the choline signal. In the suspicion of a high-grade malignancy of possible glial origin, a complete cerebellar tumor excision under general anesthesia via posterior craniotomy was performed with a surgical microscope by neuronavigation. The histological diagnosis was of a diffuse large B-cell lymphoma (DLBCL). The complete clinical-radiographical staging was negative, as well as a bone marrow biopsy. Therefore, the patient underwent chemotherapy with 4 cycles of methotrexate (MTX) and 3 of high-dose cytosine arabinoside (ARA-C). After two cycles, the patient achieved complete remission (CR). Four months after he was discharged with a diagnosis of cognitive impairment due to chemotherapy. Seventy-two months after the diagnosis, the patient is still in CR.

### 2.2. Case 2

A 65-year-old Caucasian woman with a history of dyslipidemia, alcoholism, heavy smoking, and two spontaneous miscarriages, came to our attention after she performed a CT scan showing a single lesion in the posterior cranial fossa. MRI could identify two separated lesions: one in the right hemisphere with a mass effect on the fourth ventricle and a second one in the left cerebellar peduncle. A posterior craniotomy with partial cerebellar tumor excision was performed for diagnostic porpoises. The histological analysis rendered a diagnosis of DLBCL. The patient received 4 cycles of R-MTX plus high-dose ARA-C and achieved a CR after two months. Three more months later, conditioning chemotherapy and auto-transplant of hematopoietic stem cells were successfully performed. At his last follow-up, 17 months after the diagnosis, the patient was still in CR.

### 2.3. Case 3

A 65-year-old Caucasian woman with a previous diagnosis of meningioma, treated with surgery presented with long-standing headache and nausea. A CT and MRI scan showed an expansive lesion located in the right cerebellar hemisphere close to the dentate nucleus, suspicious of malignancy. A stereotactic biopsy was performed, after a cycle of steroids, and was conclusive for a DLBCL. The staging was completely negative. The patients, therefore, underwent chemoimmunotherapy according to the MATRix scheme (Methotrexate, ARA-C, Thiotepa, and Rituximab) followed by conditioning chemotherapy and auto-transplant of autologous stem cells. Eighteen months after her diagnosis, the patient is still alive, in CR.

**Figure 1:**
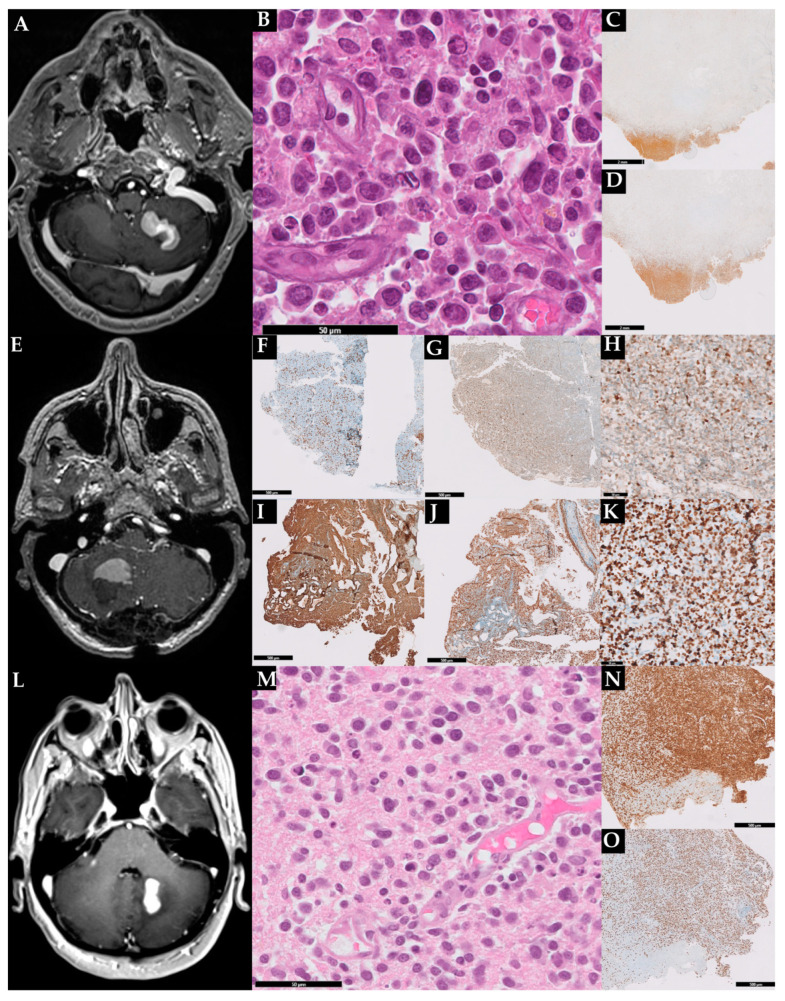
Radiological and histopathological features of the three described cases. Case 1: brain MRI showing a mass in the left cerebellar hemisphere (**A**); hematoxylin and eosin with sheets of large pleomorphic cells (**B**); immunoreactive for CD20 (**C**); the lymphoma exhibited a high proliferation index (70%) (**D**). In (**C**,**D**) is also evident a large area of necrosis. Case 2: MRI showing a mass in the right hemisphere (**E**). Histopathological analisys revealed sparse CD3+ (**F**) and Bcl2+ (**G**) T-cells and a B-cell lymphoproliferation expressing MUM1 (**H**), CD20 (**I**), and p53 (**J**); the lymphoma exhibited a high proliferation index (**K**). Case 3: brain MRI showing a mass in the right cerebellar hemisphere close to the dentate nucleus (**L**); cerebellar sample staining in HE large lymphoid cells with centralistic morphology and perivascular preservation (**M**); the lymphoma expressed PAX5 (**N**) and exhibited a high proliferation index (**O**).

The radiological and histopathological features of our three patients are further depicted in Figure 1 and summarized in Table 1 and Table 2.

### 2.4. Review of the Literature

To comprehensively review the literature on cerebellar IP-LBCL we searched PubMed, Google Scholar, Scopus, and Cochrane Library databases for articles having the following keywords in their title, abstract, or keywords: “Cerebellar”, “lymphoma”, “B-cell”, “primitive”. The last search was performed in November 2022 and the data collection and analysis were performed according to PRISMA guidelines [11].

### 2.5. Statistical Analyses

We analyzed the data gathered from the literature together with those from our patient series. We described the data in percentages based on the total number of records for each. Overall survival (OS) was calculated as the time between diagnosis and death or last follow-up and was estimated by Kaplan–Meier product-limit method. Two-sided *p*-values < 0.05 were considered statistically significant. Statistical analysis was performed using GraphPad Prism software (GraphPad Software Inc., San Diego, CA, USA, 8th version).

## 3. Results

From the first literature search, we collected 884 hits. After the exclusion of duplicates and non-relevant reports, we included in the study 14 articles spanning from 1968 to 2022, describing 23 patients (Table 1).

Counting our patients, we collected the data from 26 patients with cerebellar IP-LBCL. Of these 14 were females (male-to-female ratio of 0.86:1). The median age at diagnosis was of 57 years (ranging from 22 to 78 years). The median time from symptoms to diagnosis was 30 days.

**Figure 2:**
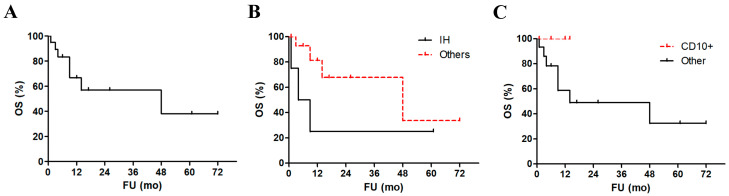
Survival analyses of cerebellar IP-LBCL patients. Overall survival of the patients’ cohort (**A**). Comparison of survival of patients with and without intracranial hypertension ((**B**), *p* = 0.03) and between those with reported CD10 expression and other cases ((**C**), *p* = 0.25).

Most patients reported at least one symptom related to cerebellar localization (ataxia, vertigo, headache, and gait disturbances, being the most common). Interestingly, intracranial hypertension was diagnosed in five patients and was associated with a significant worsening in patients’ survival at statistical analysis (*p* = 0.03, Figure 2A,B) [12,13,14]. Only two cases reported B-symptoms [15,16]. From a clinical point of view, often the cerebellar masses were suspicious for a primary or of SNC or metastasis (4 cases), a localization of tuberculosis (2 cases) vascular diseases (2 cases), and only two cases were suspected to be a lymphoma. The vast majority of patients displayed single cerebellar masses, while just 7 of them had multiple lesions (27%). Two patients received a prior diagnosis of breast cancer [17], one of atrial myxoma [18] and the last one of myasthenia gravis [19]. Complete radiographic studies, clinical examination, and bone marrow biopsies revealed a cerebellum-limited disease in all cases with no other localization, including nodal ones. However, in 2 out of the 6 analyzed cases, the liquor analysis resulted positive for neoplastic cells. Interestingly, these two patients were the same (and only) presenting with B-symptoms. No patient had a previous or concurrent history of HIV, HBV, HCV, or EBV infection. All of the other clinical findings just mentioned failed to show a significant association with patients’ survival at statistical analysis.

Histopathology reports described a monomorphic to polymorphic population of medium to large-sized lymphoid cells with scant cytoplasm, prominent nucleoli, and brisk mitotic figures. Immunostaining showed invariably a B-cell phenotype (i.e., positivity to either PAX5, CD19, CD20, and/or CD79a). We could analyze the Hans algorithm in only six cases, being in five cases (83%) germinal center B-cell phenotype lymphomas. Furthermore, in 5/6 tested cases CD10 was reported as positive and none of these patients died, despite lacking a significant prognostic advantage compared to other cases (Figure 2C). Bcl6 was positive in 2/4 cases (50%) and MUM1 in 4/4 tested cases (100%). The median proliferative index (Ki67/Mib1) was as high as 83%. Necrosis was observed in only 2 cases. FISH for MYC, BCL2, and BCL6 were performed in two, one, and three cases, respectively, and resulted invariably negative.

Histopathology reports described a monomorphic to polymorphic population of medium to large-sized lymphoid cells with scant cytoplasm, prominent nucleoli, and brisk mitotic figures. Immunostaining showed invariably a B-cell phenotype. We could analyze the Hans algorithm in only three cases. However, in 5/6 tested cases (83%) CD10 was reported as positive, suggesting a number of GC cases higher than expected in IP-LBCL. Furthermore, none of the CD10+ cases died, despite lacking a significant prognostic advantage compared to other cases (Figure 2). Bcl6 was positive in 2/4 cases (50%) and MUM1 in 4/4 tested cases (100%). The median proliferative index (Ki67/Mib1) was as high as 83%. Necrosis was observed in only 2 cases. FISH for MYC, BCL2, and BCL6 were performed in two, one, and three cases, respectively, and resulted invariably negative.

Most patients were treated with chemotherapy (78%), more commonly with methotrexate and/or cytarabine, followed by the combination of chemotherapy and radiotherapy (43%). Only two patients showed a local recurrence. Seven patients (30%) died of disease with a median time to death of 6 months, while 5 (26%) achieved a complete remission (CR). The median follow-up was of 6 months and the 5-year OS was 38% (Figure 2A).

## 4. Discussion

CNS IP-LBCL is infrequent and it represents only around 3% of all newly diagnosed primary brain tumors and up to 6% of all extranodal lymphomas [8,9]. The first cases of cerebellar IP-LBCL were described by B.E. White in 1968 [20]. This occurs only almost 40 years after the description of “primary DLBCL of the central nervous system“ by P. Bailey, who conceived it as a perivascular sarcoma of the CNS [21]. The cerebellar localization has never been considered out of the context of this last category; however, our work highlights some relevant clinical, biological, and prognostic features of this subset of cases.

The epidemiology of cerebellar and other CNS-IP-LBCL is superimposable. Both are disorders arising in people in their sixties. However, while other CNS-IP-LBCL is slightly more frequent in males, cerebellar cases are more often seen in females.

Their clinical presentation includes a raise in intracranial hypertension (e.g., headache, vision disturbances) and focal neurological deficits. Obviously, cerebellar cases generally display symptoms such as ataxia, and gait disturbances which may suggest their specific localization. Interestingly, we demonstrate that a diagnosis of intracranial hypertension in cerebellar IP-LBCL is associated with inferior survival. Moreover, the only two cases presenting with B-symptoms were associated with liquor analysis positive for neoplastic lymphocytes suggesting a possible systemic release of inflammatory cytokines.

The more common clinical suspicion was metastasis, followed by lymphoma, glial tumors, infection, and ictus. At CT scan the neoplasm appears as isodense or hyperdense masses and enhances on contrast as observed in our cases. Still, MRI is the elective and most sensitive technique for detecting IP-LBCL and most lesions are hypointense on T1-weighted images, isointense or hyperintense on T2-weighted images, and enhance moderately to markedly post contrast administration [5,14]. Sometimes a higher T2 signal can be detected around the lesion, representing tumor-associated vasogenic edema [16].

The gold standard to diagnose IP-LBCL is the stereotaxic biopsy. Cerebellar IP-LBCL were histologically diagnosed as DLBCL in all cases, as in the majority of other CNS IP-LBCL [22]. Histologically, lesions are characterized by sheets of large and more or less atypical B cells. While most IP-LBCL cases display a non-germinal center B-cell (GCB) immunophenotypical profile according to the Hans algorithm, we observed an unexpectedly high number of cases with germinal-center B-cell phenotype [23]. This is of special interest, especially considering that CD10 positivity is an index of brain dissemination of lymphoma arising outside the CNS. Despite the low number of cerebellar IP-LBCL we studied, we hypothesize that this interesting feature may be associated with the higher OS observed in these cases compared with other CNS IP-LBCL (see below). The fraction of cases reporting Bcl6 and MUM1 positivity hampered our possibility to perform any statistical analysis. The median percentage of Ki67/Mib1+ cells in cerebellar IP-LBCL was high, even though it was evaluated in a handful of cases. In two more cases, areas of necrosis were observed, corroborating the proliferative nature of these neoplasms [5]. These two last features are superimposable with CNS IP-LBCL.

From a systematic point of view, IP-LBCL is conceptually included in both the current classifications of lymphoid neoplasms (i.e., the 5th edition of the WHO classification and the International Consensus Classification (ICC)) [5,10]. The only differences are on a nomenclatorial and prudential level. Indeed, the ICC explicitly says that despite recognizing the existence of a group of extranodal lymphoma with non-GC phenotype, the unification of lymphomas arising primarily in the CNS, skin, testis, breast, adrenal glands, and intravascularly is still a matter of debate. The most critical point is the heterogeneity of these lymphomas in certain sites. Therefore, ICC authors have preferred to wait for a more comprehensive clinical, histopathological, and molecular characterization of such entities before changing the nomenclature previously adopted in the revised 4th classification of the WHO. Similarly, the 5th WHO classification states that given the rapid increase in the knowledge on these lymphomas, future classification will probably show an expansion of the IP-LBCL category, with the inclusion of other and currently distinct entities such as primary cutaneous large B-cell lymphoma, leg-type or DLBCL arising in the breast.

At clinical and radiological staging (including MRI and PET scan, lumbar puncture, bone marrow biopsy, and testicular ultrasound) all the studied cases were negative for disease spread, as observed virtually in all CNS IP-LBCL cases [3]. This was an important datum, in connection with that of CD10 positivity, and may suggest a phenotypic and biological diversity between cerebellar and other CNS IP-LBCL.

CNS IP-LBCL is considered an aggressive tumor with a high recurrence rate following treatments and a 5-year OS of 30%. Nevertheless, cerebellar cases seem to pursue a slightly less aggressive behavior, and have a higher OS (48 vs. 36 months, respectively), as we demonstrate, in statistical analysis [24].

The current therapy management for all CNS IP-LBCL consists of induction and consolidation therapy (with methotrexate, an alkylating agent, and rituximab). Still, in a variable number of cases, patients receive steroids before the biopsy. This may constitute a relevant diagnostic pitfall for lymphoma (i.e., “steroid-mitigated lymphoma”), as observed in two cases included in our review [15,25]. Even though steroid is useful in reducing the burden of symptoms, they change the radiographic and histopathological appearance potentially affecting diagnosis [26]. Moreover, in our cohort of patients, none of the patients who receive neoadjuvant steroid therapy died, compared to 40% of deaths in patients who do not receive neoadjuvant steroids. Despite this, at statistical analysis, the difference was not significant (*p* > 0.05).

The differences we highlight in our present work should be considered within some recent advances in the regional immunity of the CNS. In fact, in the last years, a growing number of papers demonstrated that the cerebellum is immunologically distinct with respect to the other CNS parts [7]. The CNS is remarkably different from peripheral organs in terms of its immunobiology as a consequence of the need to avoid tissue damage secondary to immune activation. For this reason, CNS of vertebrates have developed specialized resident immune cells (i.e., yolk sack-derived microglia) and filtering barriers against peripheral immune cells and antigens, i.e., the brain–blood barrier, the blood–CSF barrier of the choroid plexus. The CSF contains leukocytes, being T cells for the most part. However, under physiological conditions, these are not allowed to cross the blood–brain barrier. After sensing apoptotic, ischemic, or infective signals, microglia produce inflammatory mediators that may compromise the integrity of the blood–brain barrier only if there are maintained chronically, or if there is a disruptive change of the barrier itself [27]. Of importance, the breakdown of the barrier may, not only allow the entrance of peripheral immune cells but may also cause the leakage of antigens normally sequestered inside the CNS. This mechanism may be at the base of the pathogenesis of an autoimmune response against CNS autoantibodies [7].

Even though the physiological reasons are currently unknown, it has been demonstrated that the above-mentioned mechanism of immune protection of the CNS, differs among the CNS regions. Specifically, the cerebellum has a higher permeability of the blood–brain barrier of this organ as well as a higher vulnerability to inflammatory processes and different mechanisms of leukocyte trafficking given its lower expression of tight junction components such as claudin-1 and higher expression of adhesion molecules such as VCAM-1 and ICAM [7,28]. These peculiarities of the cerebellum are claimed to be the cause of its susceptibility to a variety of an increasing number of autoimmune conditions known as immune-mediated cerebellar ataxias. These are primary or secondary autoinflammatory diseases involving the cerebellar parenchyma, that presents mainly in female adults. Interestingly the etiopathogenesis of immune-mediated cerebellar ataxias seems to be related to B-cell dysfunction and age-related chronic and low-grade tissue inflammation, as well as to reduced permeability and exchange of CSF at the blood–brain barrier level [7]. The immunological specificities of the cerebellum may thus be also at the base of the different biological and prognostic features of cerebellar IP-LBCL such as the high incidence in females or the frequency of the GC B-cell phenotype. Eventually, the higher permeability of the cerebellum to peripheral immune cells (including tumor-suppressive cells) is also possibly linked to the higher OS of cerebellar IP-LBCL compared to CNS-LBCL.

## 5. Conclusions

In this work, we describe a series of cerebellar IP-LBCL and collected and analyzed the medical literature on this rare lymphoma. Doing so we highlighted many interesting epidemiological, biological, and prognostic differences between cerebellar cases and those involving other CNS structures. Indeed, cerebellar IP-LBCL is a neoplasm that affects more frequently females in their sixties and presents with symptoms related to cerebellar involvement such as ataxia or gait disturbances. All cases displayed a DLBCL morphology, possibly with a higher fraction of cases with a germinal center B-cell phenotype compared with other IP-LBCL. Finally, cerebellar IP-LBCL showed better OS survival.

All these features need to be validated in further studies on larger cohorts of patients and possibly in the context of a multi-institutional effort. Moreover, it should be determined whether and how the immunological peculiarities of the cerebellum contribute to the pathogenesis of this intriguing subset of lymphoma. This may further contribute to the understanding of the biological links between regional immunity and lymphomagenesis.

## Figures and Tables

**Table 1:** Clinical data, treatment, and outcome of 26 cases of primary large B-cell lymphomas of immune-privileged sites (including our 3 cases) diagnosed between 1968 and 2022.

	Author	Year	Sex	Age	B-Symptoms	Ataxia	Gait/ Coordination Disturbances	Headache	Intracranial Hypertension	Nausea/Vomit	Vertigo	Vision Disturbances	Number of Lesions	Neoplastic Cells at Liquor Analysis	Steroid Therapy before Biopsy	Treatment	Outcome	Follow-Up (mo)
1	White BE	1968	M	56	N	x		x			x	x	S	N	N		DOC	0
2	Chang RH	2009	M	67	N	x	x	x	x	x	x	x	M	N	N	B, Rt	DOD	4
3	Chang RH	2009	F	64	N	x	x				x		S	N	N	B, Rt	DOD	48
4	Chang RH	2009	M	62	N	x	x	x	x	x	x	x	S	N	N	B, Rt	CR	61
5	Weiqing L	2010	F	70	N					x	x		S	N	N			
6	Makhdoomi R	2011	F	45	N		x	x					S	N	N	C, Rt		0
7	Makhdoomi R	2011	M	40	N		x						S	N	N	C, Rt	DOD	14
8	Makhdoomi R	2011	F	60	N				x				M	N	N	C, Rt	DOD	9
9	Makhdoomi R	2011	F	60	N				x				S	N	N	None	DOD	1
10	Qiwu D	2011	M	60	N			x		x			S	N	N	C, Rt	LFU	6
11	Datta A	2013	F	55	Y	x				x			S	Y	Y	Rt, carmustine	CR	12
12	Bhawna J	2015	F	63	N								S	N	N	R, S, cyclophosphamide, doxorubicin, vincristine		2
13	Beraldo GL	2018	F	52	N	x					x		S	N	N	ARAC, S	DOD	3
14	Ghannam M	2018	M	71	N	x				x		x	M	N	Y	ARAC, MTX, S	SD	1
15	Harley B	2018	F	40	N			x					S	N	N	ARAC MTX	PD	6
16	Franzini A	2018	M	60	N		x		x				S	N	N		LFU	0
17	Galarza Fortuna G	2019	F	78	N	x							S	N	N	MTX, R, Rt, temozolomide	CR	14
18	Sansoni GA	2021	F	57	N	x	x			x			M	N	N	ARAC, MTX, R, Rt	PR	3
19	Sirinoglu D	2021	F	58	N			x		x			S	N	Y	MTX, S	CR	6
20	DeRon jr N	2022	M	66	Y	x							M	Y	N	MTX, S, leucovorin.	SD	
21	He M	2022	M	22	N					x		x	S	N	N	S, cyclophosphamide, epirubicin, vincristine	DOD	9
22	He M	2022	M	26	N	x					x		S	N	N	ARAC; MTX	CR	1
23	He M	2022	M	54	N								M	N	N	Rt	CR	6
24	Case n. 1	2013	M	76	N	x		x		x			S	N	N	ARAC; MTX	CR	72
25	Case n. 2	2020	F	65	N			x					S	N	N	ARAC, MTX, R	CR	26
26	Case n. 3	2021	F	65	N								M	N	N	ARAC, MTX, R	CR	17

ARAC = cytarabine, BOMES = BLNU, vincristine, methotrexate, etoposide, and methylprednisolone, C = chemotherapy, CR = complete remission, DLBCL = diffuse large B-cell lymphoma, DOC = died of other causes, DOD = died of disease, F = female, LFU = lost to follow-up, M = male, MTX = methotrexate, N = NO, PR = partial remission, R = rituximab, Rt = radiotherapy, SD = state of disease, S = steroid, Y = yes.

**Table 2:** Comparison of the clinical-pathological and prognostic features of cerebellar and CNS-IP-LBCL according to references [8,9,10].

	Cerebellar IP-LBCL	Overall CNS IP-LBCL
Age, median [range]	57 [22–78]	56 [52–70]
M:F ratio	0.86:1	1.5:1
Single lesions, %	63%	60–70%
Symptoms	Ataxia, headache, nausea	Cognitive dysfunction, focal neurological symptoms
CSF involvement, %	7.3%	7.6%
CD10+ cases, %	83%	<10%
Bcl6+ cases, %	50%	60–80%
MUM1+ cases, %	100%	90%
Median proliferation (% Ki67/Mib1+ cells)	83%	70–90%
Overall survival, months	48	36

CNS = central nervous system, F = female, IP-LBCL = primary large B-cell lymphomas of immune-privileged sites, M = male, CSF = cerebrospinal fluid.

## Data Availability

The data presented in this study are available on request from the corresponding author.
